# Hostile intruder: *Toxoplasma* holds host organelles captive

**DOI:** 10.1371/journal.ppat.1006893

**Published:** 2018-03-29

**Authors:** Isabelle Coppens, Julia D. Romano

**Affiliations:** Department of Molecular Microbiology and Immunology, Johns Hopkins University Bloomberg School of Public Health, Baltimore, Maryland, United States of America; Buffalo, UNITED STATES

## The secret weapons of *Toxoplasma*: Seclusion, secretion, and scavenging

*Toxoplasma gondii*, a human pathogen of the Apicomplexa phylum, is an obligate intracellular parasite, i.e., a microbe that must reside within a foreign cell to survive and propagate. To achieve intracellular replication, *Toxoplasma* has mastered three strategies: seclusion, secretion, and scavenging. Upon invasion, the parasite secludes itself from the host cytoplasm by forming the parasitophorous vacuole (PV), a self-made niche that protects it from host cell assaults. Within its PV, *Toxoplasma* secretes many proteins that transform the PV into a replication-competent milieu, and it subverts many host cell pathways by exporting proteins into the host territory. The parasite relentlessly scavenges nutrients from the host mammalian cytosol and organelles until egress. Hereafter, we focus on the unique properties of the *T*. *gondii* PV in relation to the scavenging of host cell–derived nutrients by the parasite.

## What are the key features of the PV?

**Fig 1:**
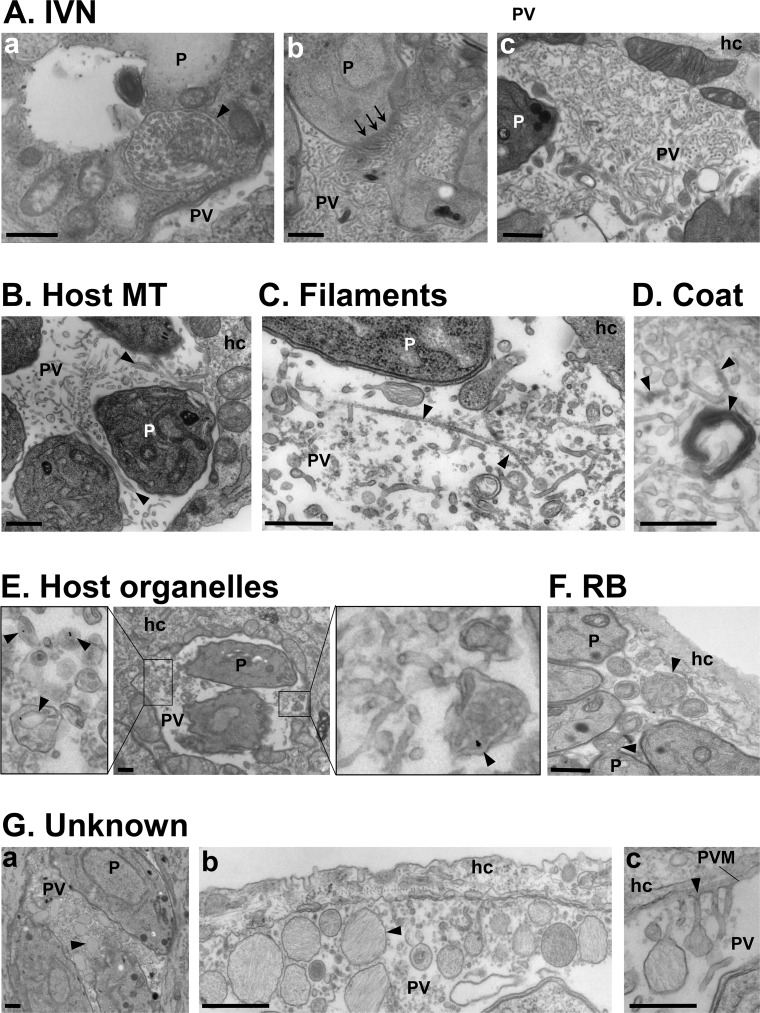
Intraluminal structures in the *Toxoplasma* PV. **(A-G)** Transmission EM of intracellular *Toxoplasma*. **(A)** Thin tubules formed by the parasite (P) 20 min post-invasion and packaged within a vesicle (panel a; arrowhead) before being discharged at the basal end of the parasite (panel b; arrows) and spread within the vacuole to form the IVN. **(B)** Host MT–based invaginations of the PV membrane (arrowheads). **(C)** Long microfilaments narrower than the IVN tubules (arrowheads). **(D)** IVN tubules and microtubular structures are coated by e-dense material. **(E)** Host endocytic organelles containing LDL-gold particles (arrowheads) surrounded by the PV membrane. **(F)** RB of the mother cells identified by discarded organelles such as the ER, either still attached to daughter cells or free in the PV lumen (arrowheads). **(G)** Unknown membrane-bound structures containing fibrillary material (arrowheads) accumulated between parasites (panel a), close to the PV membrane (panel b), or appending to the PV membrane (panel c). All scale bars, 500 nm. e-dense, electron-dense; EM, electron microscopy; ER, endoplasmic reticulum; hc, host cell; IVN, intravacuolar network; LDL, low-density lipoprotein; MT, microtubule; P, parasite; PV, parasitophorous vacuole; PVM, parasitophorous vacuole membrane; RB, residual body.

A remarkable hallmark of the PV is the presence of membranous tubules and filamentous structures in the PV lumen. Within 10–20 min post-invasion of a mammalian cell, *Toxoplasma* expels into the PV lumen an entangled network of membranous tubules 40–60 nm in diameter [1] (Fig 1A); in more recent publications, the mean diameter of these tubules tends to be 30–35 nm [2] and 28.6 ± 5 nm [3], based on measurements using super-resolution electron microscopy (EM). Many proteins discharged from secretory dense-granule (GRA) organelles localize to this intravacuolar network (IVN), and among them, GRA2 and GRA6 are critical for the maintenance of the IVN architecture [4]. During replication, the IVN expands through the salvage of lipids by the parasite from the host cell [5,6]. The IVN, containing protective antigens, plays a role in immune modulation by interfering with the major histocompatibility complex-I (MHC-1) pathway [7]. Some IVN tubules are appended to the PV membrane, likely as a result of fusion events, allowing the access of host material to the PV milieu—a point discussed later in this review [1–3,8]. The parasite further increases the permeability of the PV membrane by creating pores formed by GRA17 and GRA23, which mediate the passage of small (<1.9 kDa) solutes, i.e., host sugars or nucleotides, across this membrane [8,9]. In addition to the IVN tubules, the PV contains long actin filaments inside 50–60-nm membranous structures, thinner filamentous structures that connect the parasites and the PV membrane (Fig 1C), and many fibril-containing vesicles of unknown function (Fig 1G) [3,9]. It is unknown whether these different membranous tubules, filamentous structures, and vesicles are separate or continuous, forming a large, dynamic network inside the PV. Studies of the dynamic nature and interconnectivity of these structures are needed.

The arrangement of parasites within the PV and the connectivity of PV membranes allow the transfer of material between individual parasites and PV, respectively. Intravacuolar *Toxoplasma* tend to divide in a synchronized fashion, with two daughter cells built inside the mother parasite [10]. Parasites are arranged in a radial structure or rosette, with their basal ends attached to the residual body, a structure corresponding to the remnant of the mother cell (Fig 1F). The residual body fulfills an important role in the maintenance of the rosette conformation of the parasites [11], allowing the exchange of solutes between parasites via their basal ends, the synchronization of divisions, and an adequate orientation for egress of the parasites from the host cell [9,11,12]. The intravacuolar filamentous actin (F-actin) network, encoded by the *act1* gene, and the myosin motor MyoI, localized to the residual body, control the synchronicity of parasite divisions and mediate the transfer of material between parasites; *Toxoplasma* conditional knockouts of both *act1* and *myoI* neither form rosettes nor transfer material, and these mutants show growth defects [9,12]. In addition to intravacuolar communication, the PV membrane remarkably forms long membranous structures, named PV membrane projections (PVMP), that extend into the host cytoplasm and interconnect PV in the same host cell and even distant PV located in neighboring cells [8,13–17]. Some GRA proteins, including GRA14, are transported from one PV to another through the PVMP [14]. The nature of the materials exchanged between parasites via residual bodies and the physiological relevance of intervacuolar protein transport remain to be elucidated, but such interparasite communication underscores the importance of coordinated activities for *Toxoplasma*. Of interest, some PVMP also appear to physically interact with mammalian organelles [15,17], possibly influencing host cell functions or diverting nutrients from host organelles.

## How does the PV interface with the host cell?

The PV membrane is derived from the host cell’s plasma membrane but is distinct from a phagosomal membrane, as it does not contain host proteins susceptible to recognition and fusion with the endolysosomal system (reviewed in [18]). However, the PV is not segregated from the host cell’s endomembrane system but instead acts as a magnet by attracting many host organelles. First, host mitochondria and rough endoplasmic reticulum (ER) physically associate with the PV [19,20]; the anchorage of host mitochondria to the PV membrane is mediated by the dense-granule protein, mitochondrial association factor 1 (MAF1) [21]. Second, host endocytic structures, multivesicular bodies, Golgi ministacks, lipid droplets, and a plethora of transport vesicles associated with different Rab proteins (small monomeric Ras-like GTPases) gather around the PV, and their perivacuolar accumulation perseveres throughout infection [6,8,17,22,23].

One conundrum is what drives the tropism of host organelles to the PV. As *Toxoplasma* hijacks the host microtubule–organizing center and wraps its PV with host microtubules [22,24–26], it is plausible that the parasite governs host microtubule–based trafficking pathways, resulting in the rerouting of organelles that travel along microtubules to the PV. Alternatively, PVMP may forage and lasso host organelles to convey them to the PV vicinity. Another possibility would be the secretion of molecules by the parasite into the host cytosol that may attract mammalian organelles by creating chemical or osmotic gradients. To better understand the *Toxoplasma*–host interplay, more parasite effectors involved in host organelle interception need to be identified and functionally characterized.

**Fig 2:**
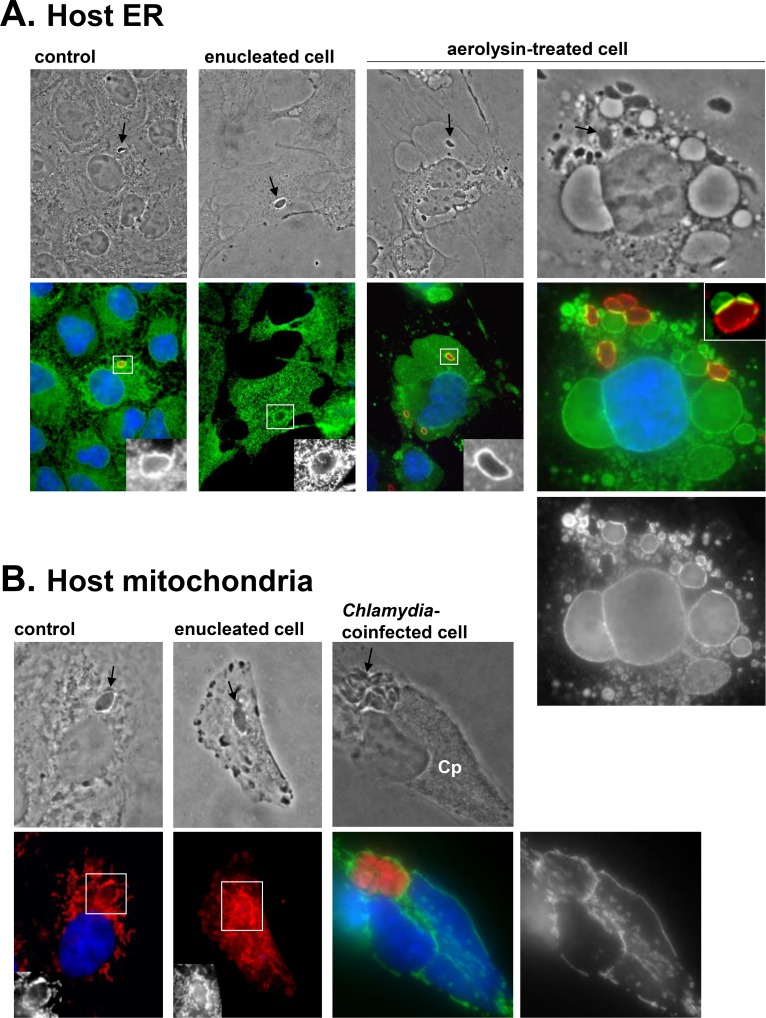
Recruitment of host organelles by *Toxoplasma* under adverse host conditions. **(A–B)** Fluorescence microscopy of host ER with anti-calnexin antibody (green in [A]) and host mitochondria with mito-Tracker (red in B) or anti-Tom20 antibody (green in [B]). Nuclei are DAPI-stained. **(A)** Host ER association with the PV (identified by arrows on phase-contrast images or stained with anti-GRA7 antibody in red) in control mammalian cells, enucleated cells, or cells treated with aerolysin, which induces the vacuolization of host ER [41]. For this last condition, Vero cells were preincubated with 0.38-nM aerolysin for 2 h and infected for 10 min without toxin, then re-exposed to 0.38-nM aerolysin for 30 min. Despite extensive deformation of the host ER with the toxin, the parasite is able to attract this organelle, as exemplified by the intense zones of contact between the PV and host ER “bubbles,” clearly visible in the inset from another aerolysin-treated cell. **(B)** Host mitochondria association with the PV (identified by arrows on phase-contrast images) in control mammalian cells, enucleated cells, or cells coinfected with RFP-*Toxoplasma* (arrow) and *Chlamydia psittaci* (Cp) for 24 h. *C*. *psittaci* is notorious for attracting host mitochondria to its inclusion [42]. These bacteria multiply faster than *Toxoplasma*, occupying a large portion of the host cytoplasm. Despite these physical constraints, *Toxoplasma* manages to attract host mitochondria to the PV to a similar extent as *Chlamydia*. anti-Tom20, mitochondrial translocase of outer membrane; Cp, *Chlamydia psittaci*; GRA7, *Toxoplasma* parasitophorous vacuole membrane protein; ER, endoplasmic reticulum; PV, parasitophorous vacuole; RFP, red fluorescent protein.

Remarkably, host organelle recruitment even occurs under adverse conditions for *Toxoplasma*. Examples include infection of cytoplasts (i.e., dying fragments of cytoplasm) [25], coinfection with microbes that attract host organelles, such as the bacterial *Chlamydia sp*. that divert host ER or mitochondria to their inclusion [27–29], or following exposure of mammalian cells to toxins that distort organelles, e.g., aerolysin-induced ER vacuolization (Fig 2A and 2B). This hints that host organelle hijacking is actively mediated by *T*. *gondii* and is part of the parasite’s program of intracellular infectivity.

Besides attracting and clustering host organelles around the PV, *Toxoplasma* selectively internalizes various host organelles into the vacuolar space. For example, host endocytic organelles (Fig 1E), multivesicular bodies, lipid droplets, and Rab vesicles—especially those involved in anterograde, recycling, and endocytic pathways—are detected inside the PV, surrounded by the PV membrane [6,8,15,17]. The PV membrane forms invaginations extending into the PV lumen. Some invaginations are formed following fusion of an IVN tubule with the vacuolar membrane or are generated by host microtubules poking the PV membrane (Fig 1B). Host organelles use either type of invagination of the PV membrane as conduits, resulting in their sequestration into the vacuole, based on EM observations. These PV invaginations that generate gateways for host material intake are likely to be selective, excluding some host organelles such as ER elements [30] or peroxisomes [15]. This suggests the presence of parasite effectors involved in host protein or organelle recognition on these PV membrane invaginations. Sometimes visible on the surface of these invaginations is a dynamin-like coat (Fig 1D) that induces the constriction of the invagination and contributes to the confinement of host organelles inside the vacuole [15]. Furthermore, parasites with a defective IVN *(Δgra2* and *Δgra2Δgra6* mutants) scavenge fewer host Rab vesicles, endosomes, and lipid droplets, suggesting a role for the IVN in the internalization of host-derived materials [6,8].

Intravacuolar host organelles are further enwrapped by the IVN and concentrated at the middle of the rosette formed by the parasite [6,8]. An intriguing question is how the IVN and host organelles move inside the PV. Possibly, these structures move in a directed fashion inside the PV along actin filaments that reside within long and dynamic membranous tubules [9]. Alternatively, a cytoplasmic streaming-like motion (cyclosis) may circulate in the PV lumen, displacing host organelles by Brownian motion, with no pattern of directionality. Super-resolution live-cell imaging of the PV would provide central information about the dynamics of host organelles during their penetration into the PV, intravacuolar movement, and fate in the vacuolar space.

## Why does the PV recruit or internalize host organelles?

A central question is why *Toxoplasma* attracts and consumes so many different organelles. Possible reasons include the control of the immune response, the scavenging of nutrients, the neutralization of harmful host organelle functions, or the interception of host trafficking pathways for the parasite’s benefit.

The parasite may intercept host organelles to control the immune response. For example, the association of the host ER with the PV membrane may be advantageous for the parasite to deliver antigens from the PV lumen to the host ER [31]. Parasite antigens may then be exported from the host ER into the host cytosol and trimmed by the ER-associated protein degradation (ERAD) system into peptides that enter the host ER for association with the MHC-I complex and CD8^+^ (cytotoxic) T-cell cross-priming. The outcome of CD8^+^ T-cell activation by *Toxoplasma* antigen is the secretion of interferon gamma (IFNγ), which allows the parasite to encyst and persist in the host.

Surely, the parasite harnesses host organelles to take advantage of their profuse nutrient content. The parasite scavenges host sphingolipids from Golgi-derived vesicles because dominant negative mutants of Rab14 and Rab43 lead to a decrease in host-derived sphingolipid associated with the PV [17]. In another example, *Toxoplasma* salvages host cholesterol from endolysosomes and host fatty acids from lipid droplets [6,15]. In fact, parasite replication is decreased in host cells with reduced triacylglyceride lipolysis and fatty acid catabolism (two enzymatic functions associated with lipid droplets), with depleted lipid droplets or with impaired cholesterol egress from host lysosomes [6,22]. Of note, host Rab vesicles involved in the recycling pathway, e.g., Rab4 and Rab11, are extensively hijacked by *Toxoplasma*. Possibly, interfering with the host recycling pathway, e.g., the fusion of recycling endosomes with the plasma membrane, may ensure the longer retention of nutrients in the host cell, an advantage for the parasite, by blocking the export of the contents of recycling endosomes from the infected cell. Additionally, the static zones of close apposition between the PV membrane and host ER and mitochondria may function as membrane contact sites (MCS), allowing nonvesicular trafficking of molecules from these organelles to the PV lumen. For instance, host mitochondria are the source of lipoate for *Toxoplasma*, possibly making their attachment at the PV membrane an efficient mechanism to procure lipoate [32].

Intravacuolar sequestration of host cell structures may also be a protective mechanism developed by the parasite to neutralize harmful host organellar functions by controlling the abundance and distribution of host organelles. Host lipid droplets containing inflammatory mediators may be harnessed by *Toxoplasma* to control pro-inflammatory responses, thus prolonging the survival of the parasite within the host cell. In activated macrophages and neutrophils, half of the PV undergo lysosomal degradation; by preventively engulfing host degradative organelles into the PV, the parasite might reduce the frequency of fatal events involving the fusion of host endolysosomes with the PV.

Finally, *Toxoplasma* may intercept many mammalian trafficking pathways to override its host cell and disrupt intrinsic functions, e.g., pathways inducing cell death that function in host defense [33]. To this point, many GRA proteins are exported to the host cell to interact with mammalian proteins (reviewed in [34]). For most of them, a specific role has not yet been assigned, but the diversity of these extravacuolar GRA proteins may reflect the large number of different hosts infected by *Toxoplasma*.

## How does *Toxoplasma* internalize host macromolecules sequestered into the PV?

Within the PV, host organelles are trapped within IVN tubules or enwrapped by them [6,8,17], raising the question as to how the parasite accesses their content. A membrane-damaging phospholipase, lecithin:cholesterol acyltransferase (LCAT), is secreted by dense granules and localizes to the IVN [8,37]. This enzyme may be involved in the degradation of host intravacuolar organelles, because parasites overexpressing LCAT contain fewer host Rab11 vesicles [8]. In fact, parasites ablated for *lcat* also contain fewer lipids originating from host lipid droplets [6], suggesting that host organelles in the PV are processed to liberate and make available to the parasite their content. How *Toxoplasma* controls the lipolytic activities of LCAT on its own membranes needs to be clarified.

In addition to host organelles and vesicles, *T*. *gondii* internalizes host cytosolic proteins, such as GFP or mCherry, into the PV [30]. The entry mechanism into the PV for host cytosolic proteins is unknown, but PV membrane invaginations may act as gateways for host proteins present in the cytosol, because mutant parasites lacking an IVN internalize fewer host cytosolic proteins [30].

It remains to be determined how the parasite internalizes into its cell body host cytosolic proteins or organelles from the PV lumen. In fact, *Toxoplasma* is an intriguing organism regarding endocytosis, as until recently, no internalization gateways had been identified. The *Toxoplasma* genome only encodes a few homologues of endocytic machinery, e.g., AP2 adaptor complex, and the light and heavy chains of clathrin, but none are expressed at the plasma membrane and no clathrin-coated pits have been observed [36]. A single micropore, a small cup-shaped 20-nm pit of the plasma membrane, has been observed, but this invagination is rather static, which precludes a function in endocytosis [37]. Our studies on lipid uptake by *Toxoplasma*, however, had fortuitously led to the identification of endocytic structures [4]. *Toxoplasma* avidly scavenges fatty acids and upon exposure to excess (20-times) oleic acid, a large invagination of the parasite’s plasma membrane is observed [6]. The invagination, located at the parasite’s anterior end, has a narrow neck (diameter of about 250 nm) and is clearly distinct from the micropore. Coating the invagination’s cytoplasmic surface is a radiating, bristlelike structure, reminiscent of the coat on mammalian endocytic structures, e.g., clathrin. The content of the invaginations and of several cytosolic vesicles is morphologically similar to material present in the PV milieu, indicating a connection between these structures and the PV lumen and, thus, an endocytic event. The sole detection of these invaginations upon supplementation of oleic acid suggests that these processes may be particularly fast, thus impeding the trackability of these endocytic structures under standard culture conditions.

*Toxoplasma* is equipped to digest host cytosolic and organelle-derived proteins (e.g., GFP, mCherry, GFP-Rab11) in an acidified organelle named vacuolar compartment (VAC), which contains enzymes including the endopeptidase cathepsin L (CPL) [30]. Accumulation of host cytosolic GFP and GFP-Rab11 in VAC could only be observed in CPL–deficient parasites, indicative of a fast degradation process in wild-type parasites [8,30]. Interestingly, VAC proteolytic activity is required for *Toxoplasma* persistence in animals [38]. CPL-deficient parasites form autophagosomes that associate with VAC and contain undigested organelles. Further investigations into the nature of endocytic organelles containing host material and the fate of endocytosed macromolecules in wild-type parasites would be central to understanding how *Toxoplasma* feeds itself.

## Concluding remarks

The evolutionary strategy of adopting a sequestered lifestyle within specialized vacuoles over an exposed lifestyle in the nutrient-rich cytoplasm likely compromises optimal growth in favor of immune surveillance evasion. Overall, the *Toxoplasma* PV is a multifunctional, organized compartment that supports parasite development at many levels, from synchronized divisions to nutrient supply, and the integrity of the PV is central to pathogenicity. Feeding on host organelles by attracting them to the PV or engulfing them intact inside the vacuole is a strategy shared by many pathogens, including the malaria parasite [39] and chlamydial bacteria [40]. It represents a safe, alternative strategy that circumvents the need for membrane fusion with harmful mammalian organelles when delivering large amounts of food. The identification of critical determinants, i.e., the PV effectors and the co-opted host cell pathways that supply nutrients and essential metabolites, will not only enhance our understanding of parasitic strategies but also offer novel therapeutic avenues.

## References

[1] Sibley LD, Niesman IR, Parmley SF, Cesbron-Delauw MF. Regulated secretion of multi-lamellar vesicles leads to formation of a tubulo-vesicular network in host-cell vacuoles occupied by *Toxoplasma gondii*. J Cell Sci. 1995;108 (Pt 4): 1669–1677. 7615684 10.1242/jcs.108.4.1669

[2] Magno RC, Lemgruber L, Vommaro RC, De Souza W, Attias M. Intravacuolar network may act as a mechanical support for *Toxoplasma gondii* inside the parasitophorous vacuole. Microsc Res Tech. 2005;67: 45–52. doi: 10.1002/jemt.20182 16025490 10.1002/jemt.20182

[3] De Souza W, Attias M. New views of the *Toxoplasma gondii* parasitophorous vacuole as revealed by Helium Ion Microscopy (HIM). J Struct Biol. 2015;191: 76–85. doi: 10.1016/j.jsb.2015.05.003 26004092 10.1016/j.jsb.2015.05.003

[4] Mercier C, Dubremetz J-F, Rauscher B, Lecordier L, Sibley LD, Cesbron-Delauw M-F. Biogenesis of nanotubular network in *Toxoplasma* parasitophorous vacuole induced by parasite proteins. Mol Biol Cell. 2002;13: 2397–2409. doi: 10.1091/mbc.E02-01-0021 12134078 10.1091/mbc.E02-01-0021 PMC117322

[5] Caffaro CE, Boothroyd JC. Evidence for host cells as the major contributor of lipids in the intravacuolar network of *Toxoplasma*-infected cells. Eukaryotic Cell. 2011;10: 1095–1099. doi: 10.1128/EC.00002-11 21685319 10.1128/EC.00002-11 PMC3165450

[6] Nolan SJ, Romano JD, Coppens I. Host lipid droplets: An important source of lipids salvaged by the intracellular parasite *Toxoplasma gondii*. PLoS Pathog. 2017;13: e1006362 doi: 10.1371/journal.ppat.1006362 28570716 10.1371/journal.ppat.1006362 PMC5469497

[7] Lopez J, Bittame A, Massera C, Vasseur V, Effantin G, Valat A, et al Intravacuolar Membranes Regulate CD8 T Cell Recognition of Membrane-Bound *Toxoplasma gondii* Protective Antigen. Cell Rep. 2015;13: 2273–2286. doi: 10.1016/j.celrep.2015.11.001 26628378 10.1016/j.celrep.2015.11.001

[8] Romano JD, Nolan SJ, Porter C, Ehrenman K, Hartman EJ, Hsia R-C, et al The parasite *Toxoplasma* sequesters diverse Rab host vesicles within an intravacuolar network. J Cell Biol. 2017;12: jcb.201701108. doi: 10.1083/jcb.201701108 10.1083/jcb.201701108 PMC5716271 29070609

[9] Periz J, Whitelaw J, Harding C, Gras S, Del Rosario Minina MI, Latorre-Barragan F, et al *Toxoplasma gondii* F-actin forms an extensive filamentous network required for material exchange and parasite maturation. Elife. 2017;6: e24119 doi: 10.7554/eLife.24119 28322189 10.7554/eLife.24119 PMC5375643

[10] Francia ME, Striepen B. Cell division in apicomplexan parasites. Nat Rev Micro. 2014;12: 125–136. doi: 10.1038/nrmicro3184 24384598 10.1038/nrmicro3184

[11] Muñiz-Hernández S, Carmen MGD, Mondragón M, Mercier C, Cesbron MF, Mondragón-González SL, et al Contribution of the residual body in the spatial organization of *Toxoplasma gondii* tachyzoites within the parasitophorous vacuole. J Biomed Biotechnol. 2011;2011: 473983 doi: 10.1155/2011/473983 22190852 10.1155/2011/473983 PMC3228691

[12] Frénal K, Jacot D, Hammoudi P-M, Graindorge A, Maco B, Soldati-Favre D. Myosin-dependent cell-cell communication controls synchronicity of division in acute and chronic stages of *Toxoplasma gondii*. Nature Communications 2017;8: ncomms15710. doi: 10.1038/ncomms15710 28593938 10.1038/ncomms15710 PMC5477499

[13] Dubremetz JF, Achbarou A, Bermudes D, Joiner KA. Kinetics and pattern of organelle exocytosis during *Toxoplasma gondii*/host-cell interaction. Parasitol Res. 1993;79: 402–408. 8415546 10.1007/BF00931830

[14] Rome ME, Beck JR, Turetzky JM, Webster P, Bradley PJ. Intervacuolar Transport and Unique Topology of GRA14, a Novel Dense Granule Protein in *Toxoplasma gondii*. Infect Immun. 2008;76: 4865–4875. doi: 10.1128/IAI.00782-08 18765740 10.1128/IAI.00782-08 PMC2573327

[15] Coppens I, Dunn JD, Romano JD, Pypaert M, Zhang H, Boothroyd JC, et al *Toxoplasma gondii* sequesters lysosomes from mammalian hosts in the vacuolar space. Cell. 2006;125: 261–274. doi: 10.1016/j.cell.2006.01.056 16630815 10.1016/j.cell.2006.01.056

[16] Lige B, Romano JD, Bandaru VVR, Ehrenman K, Levitskaya J, Sampels V, et al Deficiency of a Niemann-Pick, type C1-related protein in *Toxoplasma* is associated with multiple lipidoses and increased pathogenicity. PLoS Pathog. 2011;7: e1002410 doi: 10.1371/journal.ppat.1002410 22174676 10.1371/journal.ppat.1002410 PMC3234224

[17] Romano JD, Sonda S, Bergbower E, Smith ME, Coppens I. *Toxoplasma gondii* salvages sphingolipids from the host Golgi through the rerouting of selected Rab vesicles to the parasitophorous vacuole. Mol Biol Cell. 2013;24: 1974–1995. doi: 10.1091/mbc.E12-11-0827 23615442 10.1091/mbc.E12-11-0827 PMC3681701

[18] Clough B, Frickel E-M. The *Toxoplasma* Parasitophorous Vacuole: An Evolving Host-Parasite Frontier. Trends Parasitol. 2017;33: 473–488. doi: 10.1016/j.pt.2017.02.007 28330745 10.1016/j.pt.2017.02.007

[19] Melo EJ, De Souza W. Relationship between the host cell endoplasmic reticulum and the parasitophorous vacuole containing *Toxoplasma gondii*. Cell Struct Funct. 1997;22: 317–323. 9248995 10.1247/csf.22.317

[20] Sinai AP, Webster P, Joiner KA. Association of host cell endoplasmic reticulum and mitochondria with the *Toxoplasma gondii* parasitophorous vacuole membrane: a high affinity interaction. J Cell Sci. 1997;110 (Pt 17): 2117–2128. 9378762 10.1242/jcs.110.17.2117

[21] Pernas L, Adomako-Ankomah Y, Shastri AJ, Ewald SE, Treeck M, Boyle JP, et al *Toxoplasma* effector MAF1 mediates recruitment of host mitochondria and impacts the host response. PLoS Biol. 2014;12: e1001845 doi: 10.1371/journal.pbio.1001845 24781109 10.1371/journal.pbio.1001845 PMC4004538

[22] Coppens I, Sinai AP, Joiner KA. *Toxoplasma gondii* exploits host low-density lipoprotein receptor-mediated endocytosis for cholesterol acquisition. J Cell Biol. 2000;149: 167–180. 10747095 10.1083/jcb.149.1.167 PMC2175092

[23] Hu X, Binns D, Reese ML. The coccidian parasites *Toxoplasma* and *Neospora* dysregulate mammalian lipid droplet biogenesis. J Biol Chem. 2017;292: 11009–11020. doi: 10.1074/jbc.M116.768176 28487365 10.1074/jbc.M116.768176 PMC5491784

[24] Melo EJ, Carvalho TM, De Souza W. Behaviour of microtubules in cells infected with *Toxoplasma gondii*. Biocell. 2001;25: 53–59. 11387877

[25] Romano JD, Bano N, Coppens I. New host nuclear functions are not required for the modifications of the parasitophorous vacuole of *Toxoplasma*. Cell Microbiol. 2008;10: 465–476. doi: 10.1111/j.1462-5822.2007.01061.x 17970763 10.1111/j.1462-5822.2007.01061.x

[26] Walker ME, Hjort EE, Smith SS, Tripathi A, Hornick JE, Hinchcliffe EH, et al *Toxoplasma gondii* actively remodels the microtubule network in host cells. Microbes Infect. 2008;10: 1440–1449. doi: 10.1016/j.micinf.2008.08.014 18983931 10.1016/j.micinf.2008.08.014 PMC2765197

[27] Romano JD, de Beaumont C, Carrasco JA, Ehrenman K, Bavoil PM, Coppens I. A novel co-infection model with *Toxoplasma* and *Chlamydia trachomatis* highlights the importance of host cell manipulation for nutrient scavenging. Cell Microbiol. 2013;15: 619–646. doi: 10.1111/cmi.12060 23107293 10.1111/cmi.12060 PMC3625693

[28] Romano JD, de Beaumont C, Carrasco JA, Ehrenman K, Bavoil PM, Coppens I. Fierce competition between *Toxoplasma* and *Chlamydia* for host cell structures in dually infected cells. Eukaryot Cell. 2013;12: 265–277. doi: 10.1128/EC.00313-12 23243063 10.1128/EC.00313-12 PMC3571308

[29] Romano JD, Coppens I. Host Organelle Hijackers: a similar modus operandi for *Toxoplasma gondii* and *Chlamydia trachomatis*: co-infection model as a tool to investigate pathogenesis. Pathogens Dis. 2013;69: 72–86. doi: 10.1111/2049-632X.12057 23821471 10.1111/2049-632X.12057 PMC3808458

[30] Dou Z, McGovern OL, Di Cristina M, Carruthers VB. *Toxoplasma gondii* Ingests and Digests Host Cytosolic Proteins. MBio. 2014;5: e01188–14–e01188–14. doi: 10.1128/mBio.01188-14 25028423 10.1128/mBio.01188-14 PMC4161261

[31] Goldszmid RS, Coppens I, Lev A, Caspar P, Mellman I, Sher A. Host ER-parasitophorous vacuole interaction provides a route of entry for antigen cross-presentation in *Toxoplasma gondii*-infected dendritic cells. J Exp Med. 2009;206: 399–410. doi: 10.1084/jem.20082108 19153244 10.1084/jem.20082108 PMC2646567

[32] Crawford MJ, Thomsen-Zieger N, Ray M, Schachtner J, Roos DS, Seeber F. *Toxoplasma gondii* scavenges host-derived lipoic acid despite its de novo synthesis in the apicoplast. EMBO J. 2006;25: 3214–3222. doi: 10.1038/sj.emboj.7601189 16778769 10.1038/sj.emboj.7601189 PMC1500979

[33] Lüder CGK, Gross U. Apoptosis and Its Modulation During Infection with *Toxoplasma gondii*: Molecular Mechanisms and Role in Pathogenesis Role of Apoptosis in Infection. Berlin/Heidelberg: Springer, Berlin, Heidelberg; 2005 pp. 219–237. doi: 10.1007/3-540-27320-4_10 10.1007/3-540-27320-4_10 15791958

[34] Hakimi M-A, Olias P, Sibley LD. *Toxoplasma* Effectors Targeting Host Signaling and Transcription. Clin Microbiol Rev. 2017;30: 615–645. doi: 10.1128/CMR.00005-17 28404792 10.1128/CMR.00005-17 PMC5475222

[35] Pszenny V, Ehrenman K, Romano JD, Kennard A, Schultz A, Roos DS, et al A Lipolytic Lecithin:Cholesterol Acyltransferase Secreted by *Toxoplasma* Facilitates Parasite Replication and Egress. J Biol Chem. 2016;291: 3725–3746. doi: 10.1074/jbc.M115.671974 26694607 10.1074/jbc.M115.671974 PMC4759155

[36] Field MC, Gabernet-Castello C, Dacks JB. Reconstructing the evolution of the endocytic system: insights from genomics and molecular cell biology. Adv Exp Med Biol. 2007;607: 84–96. doi: 10.1007/978-0-387-74021-8_7 17977461 10.1007/978-0-387-74021-8_7

[37] Nichols BA, Chiappino ML, Pavesio CE. Endocytosis at the micropore of *Toxoplasma gondii*. Parasitol Res. 1994;80: 91–98. 8202461 10.1007/BF00933773

[38] Di Cristina M, Dou Z, Lunghi M, Kannan G, Huynh M-H, McGovern OL, et al *Toxoplasma* depends on lysosomal consumption of autophagosomes for persistent infection. Nat Microbiol. 2017;2: 17096 doi: 10.1038/nmicrobiol.2017.96 28628099 10.1038/nmicrobiol.2017.96 PMC5527684

[39] Coppens I. How *Toxoplasma* and malaria parasites defy first, then exploit host autophagic and endocytic pathways for growth. Curr Opin Microbiol. 2017;40: 32–39. doi: 10.1016/j.mib.2017.10.009 29102900 10.1016/j.mib.2017.10.009

[40] Bastidas RJ, Elwell CA, Engel JN, Valdivia RH. Chlamydial intracellular survival strategies. Cold Spring Harb Perspect Med. 2013;3: a010256 doi: 10.1101/cshperspect.a010256 23637308 10.1101/cshperspect.a010256 PMC3633179

[41] Abrami L, Fivaz M, Glauser PE, Parton RG, van der Goot FG. A pore-forming toxin interacts with a GPI-anchored protein and causes vacuolation of the endoplasmic reticulum. J Cell Biol. 1998;140: 525–540. 9456314 10.1083/jcb.140.3.525 PMC2140172

[42] Matsumoto A, Bessho H, Uehira K, Suda T. Morphological studies of the association of mitochondria with chlamydial inclusions and the fusion of chlamydial inclusions. J Electron Microsc (Tokyo). 1991;40: 356–363. 1666645

