# Correction: Hostile intruder: *Toxoplasma* holds host organelles captive

**DOI:** 10.1371/journal.ppat.1007018

**Published:** 2018-04-24

**Authors:** Isabelle Coppens, Julia D. Romano

Two references were omitted from the reference list. Reference 9 should be: Schwab JC, Beckers CJ, Joiner KA. The parasitophorous vacuole membrane surrounding intracellular Toxoplasma gondii functions as a molecular sieve. Proc Natl Acad Sci USA. 1994; 91: 509–513. PMID: 8290555

Reference 10 should be: Gold DA, Kaplan AD, Lis A, Bett GC, Rosowski EE, Cirelli KM, et al. The Toxoplasma dense granule proteins GRA17 and GRA23 mediate the movement of small molecules between the host and the parasitophorous vacuole. Cell Host Microbe. 2015; 17: 642–652. doi:https://doi.org/10.1016/j.chom.2015.04.003

As a result of this omission, the reference list is out of order. Reference 9 in the list should be reference 11, reference 10 should be reference 12, reference 11 should be reference 13, and so on, as far as reference 42, which should be reference 44.
